# Metallothionein Genes are Highly Expressed in Malignant Astrocytomas and Associated with Patient Survival

**DOI:** 10.1038/s41598-019-41974-9

**Published:** 2019-04-01

**Authors:** Bernadeta Masiulionytė, Indrė Valiulytė, Arimantas Tamašauskas, Daina Skiriutė

**Affiliations:** Laboratory of Molecular Neurooncology, Neuroscience Institute, Medical Academy, Lithuanian University of Health Sciences, Eiveniu str. 4, Kaunas, LT-50161 Lithuania

## Abstract

Gliomas are heterogeneous, primary brain tumours that originate from glial cells. The main type of gliomas is astrocytomas. There are four grades (I-IV) of astrocytoma malignancy. Astrocytoma grade IV known as glioblastoma multiforme (GBM) is the most common and aggressive type of astrocytic gliomas. Metallothioneins (MT) are low molecular weight, cysteine rich proteins encoded by a family of metallothionein (*MT*) genes. *MT* genes play a crucial role in carcinogenesis of diverse malignancies. We proposed *MT* genes as prognostic markers for malignant astrocytoma. *MT1A*, *MT1E*, *MT1X*, *MT2*, *MT3* gene expression was elevated in grade IV astrocytomas (glioblastomas) as compared to astrocytomas grade I-III. Statistically significant differences were reached for *MT1A* and *MT2* genes (Mann-Whitney test, p < 0.05). High *MT1A*, *MT1X*, *MT2*, *MT3* genes expression was associated with shorter patient survival (Log-rank test, p < 0.05). *MT1A* gene promoter methylation was decreased in glioblastoma (57.6%) while the gene was highly methylated in grade II-III astrocytoma (from 66.7% to 83.3%) and associated with better patient survival (p < 0.05). *MT1A* gene methylation showed a trend of being associated with higher mRNA expression level in astrocytomas. Increased *MT* genes expression in grade IV astrocytomas as compared to I-III grade astrocytomas could be associated with malignant tumour behaviour and progression.

## Introduction

Gliomas are one of the most common groups of heterogeneous, neuroepithelial primary tumours of the central nervous system (CNS) that arise from glial cells^1,2^. There are several types of glioma that are named according to the cell type they originate from. One of the most common gliomas is astrocytomas^3^. Since 2016 the World Health Organization (WHO) has been classifying astrocytic gliomas into four grades of malignancy (grade I-IV astrocytoma) according to histological features such as necrosis, microvascular proliferation, mitoses, abnormal cells and also molecular features such as *IDH* mutation, 1p/19q deletion or *ATRX* mutation in addition to histology^2,4^. The most aggressive type of astrocytoma is grade IV astrocytoma or glioblastoma (GBM). GBM is the most lethal, aggressive type of CNS tumours. GBM is characterized by histological and molecular heterogeneity. Also, GBM is highly invasive and vascularized brain tumour resistant to treatment. The median patient survival time is about 15 months and less than 5% survive for 5 years^5^. The development of gliomas is associated with genetic and epigenetic alterations that result in the repression of tumour suppressor genes or the activation of oncogenes in the normal brain cells^6,7^.

Metallothioneins (MT) are the group of low molecular weight, metal binding proteins that are prevalent in many organisms^8^. There are 16 isoforms of MTs that are divided into 4 classes – MT1 – MT4^9^. MT proteins are encoded by metallothionein (*MT*) gene family that is located on chromosome 16q13^8,9^. MT2 – MT4 proteins are encoded by a single gene while MT1 is encoded by a set of 13 genes (*MT1A, -1B, -1C, -1D, -1E, -1F, -1G, -1I, -1J, -1L, -1M, -1X*)^8^. *MTs* are involved in tumour biology such as proliferation, differentiation, apoptosis, and angiogenesis^10^. Also, *MT* gene expression alterations are associated with carcinogenesis and with aggressive tumour behaviour and even chemotherapy resistance^11^. However, the expression of *MT* genes is not specific to any particular cancer type^12^. For example, *MT3* gene has been determined as specific to brain tissue but also it is expressed in kidney, renal carcinoma and bladder cancer^12–14^. Despite this, the importance of *MT3* expression changes in tumour is not understood yet. The expression level may depend on the differentiation of tumour, proliferation or gene mutations, for example, *p53*^12^. For instance, *MT* gene expression was associated with tumour grade and cell proliferation rate in germ cell carcinoma and breast cancer^12,15^. There is not enough information about *MT* gene expression changes in different grade gliomas. It is known that *MT* gene expression in glioblastoma tumour samples was related to shorter patients survival^16^. Thus, it is important to clarify the importance of MTs expression level change during malignant progression. The purpose of this study was to determine *MT* genes mRNA expression level in different grade glioma tumours and to disclose gene expression associated promoter methylation in gliomas.

## Results

### Associations of MT gene level and patient clinical characteristics

For the association analysis between mRNA expression and patient clinical characteristics, the mRNA expression level was assigned to “low expression” and “high expression” groups according to the median value. The mRNA expression below the median was designated as low expression and values above the median as high expression. First, we analyzed the association between *MT* mRNA expression groups and patients gender. There were no significant associations for *MT1E, MT1X* genes while *MT1A, MT2, MT3* genes showed statistically significant expression differences in males and females (Chi-square test, p < 0.05). Low mRNA expression for *MT1A*, *MT2*, and *MT3* genes was determined more frequently for man as compared to woman (39.2% vs 11.8%, 41.8% vs. 9.1%, 39.2% vs. 19.6% respectively) (Chi-square test, p < 0.05). Analysis of *MT* expression in patient age groups have shown that higher *MT1A, MT2* and *MT3* genes expression level was significantly associated with older patient age (>50 years) (Chi-square test, p < 0.05) (data not shown).

### *MT1A, MT1E, MT1X, MT2, MT3* expression increases in glioblastoma

**Figure 1:**
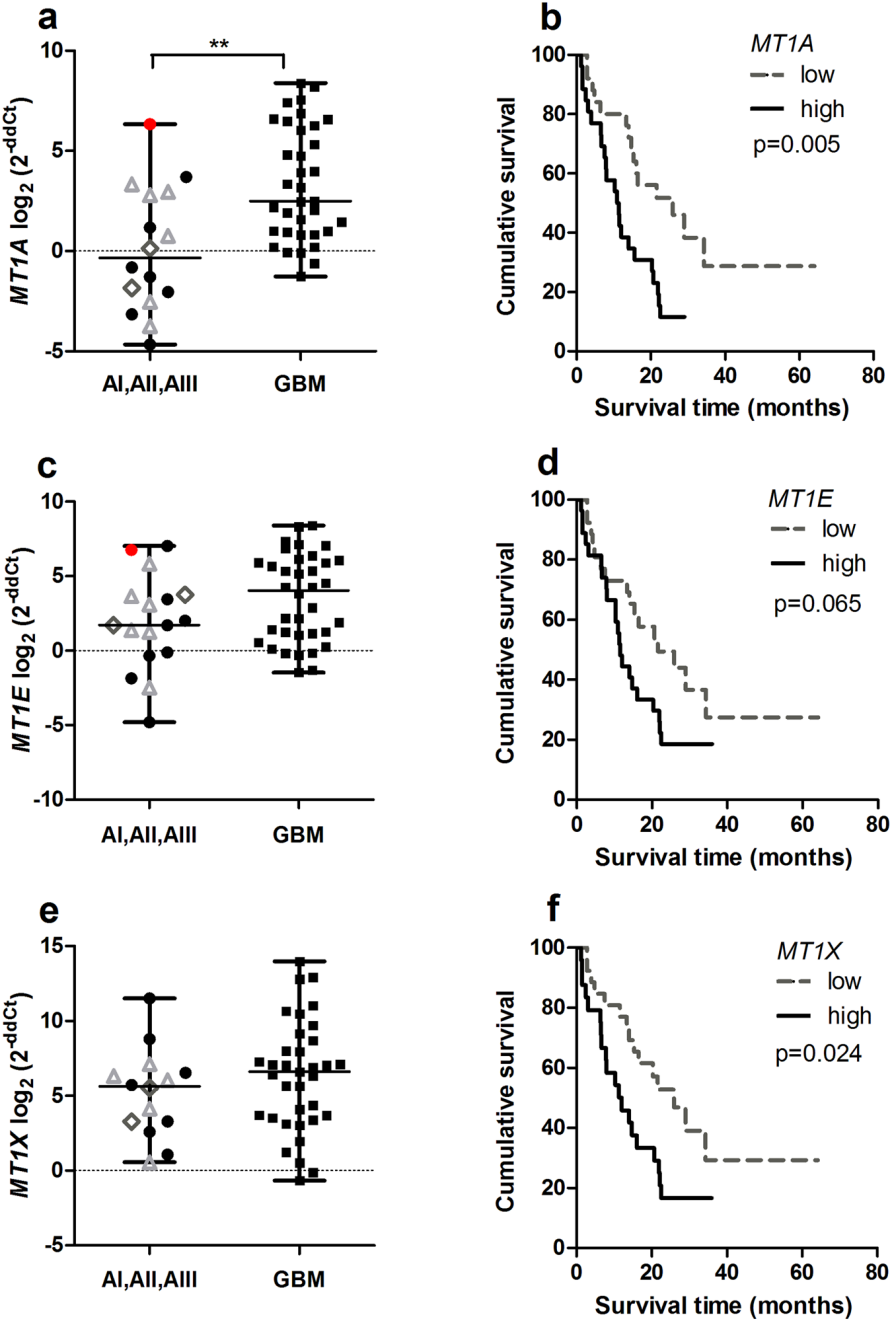
*MT1A, MT1E* and *MT1X* gene mRNA expression in different malignancy astrocytomas and expression effect on survival. (**a**) *MT1A* gene expression level in grade I-III astrocytoma and glioblastoma (GBM) (Mann-Whitney test, **p < 0.005). (**b**) Kaplan-Meier overall survival curves according to low and high *MT1A* gene expression groups (log-rank test: χ^2^ = 6.400, df = 1, p = 0.011). (**c**) *MT1E* gene expression level in grade I-III astrocytoma and glioblastoma (GBM) (Mann-Whitney test, p > 0.05). (**d**) Kaplan-Meier overall survival curves according to low and high *MT1E* gene expression groups (log-rank test: χ^2^ = 3.410, df = 1, p = 0.065). (**e**) *MT1X* gene expression level in grade I-III astrocytoma and glioblastoma (GBM) (Mann-Whitney test, p > 0.05). (**f**) Kaplan-Meier overall survival curves according to low and high *MT1X* gene expression groups (log-rank test: χ^2^ = 4.672, df = 1, p = 0.031). The middle line on the scatter plot graph represents the median value, whiskers – the lowest and the highest values. Graph symbols represent different astrocytoma grade: grade I astrocytoma – grey rhombus, grade II astrocytoma – black round, gemistocytic astrocytoma - red dot, grade III astrocytoma – grey triangle, GBM - black rectangular. Gene expression level presented as log_2_(2^−ddCt^).

**Figure 2:**
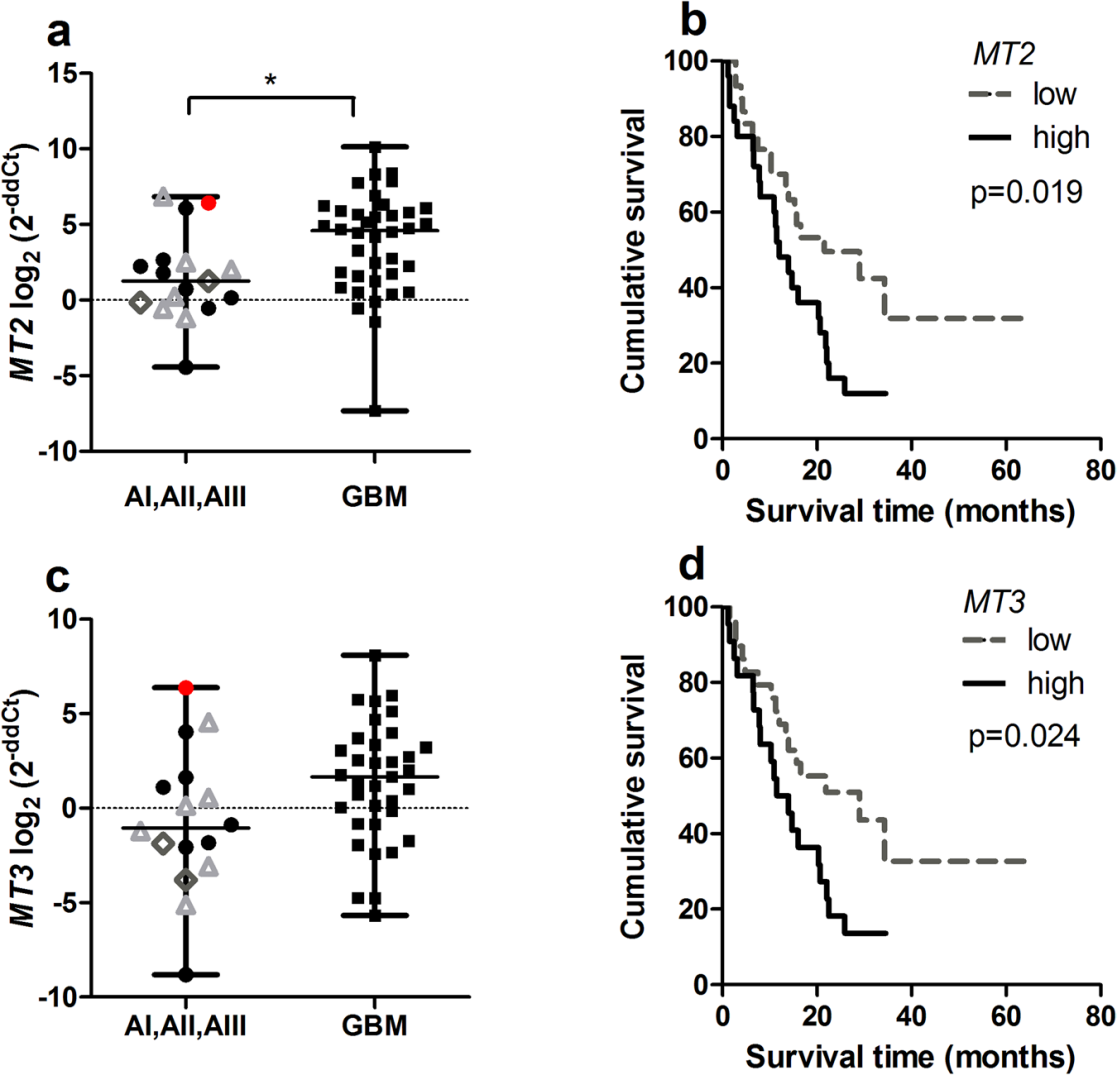
*MT2* and *MT3* gene mRNA expression in different malignancy astrocytomas and Kaplan-Meier survival curves. (**a**) *MT2* gene expression level in grade I-III astrocytoma and glioblastoma (GBM) (Mann-Whitney test, *p < 0.050). (**b**) Kaplan-Meier overall survival curves according to low and high *MT2* gene expression groups (log-rank test: χ^2^ = 8.542, df = 1, p = 0.003). (**c**) *MT3* gene expression level in grade I-III astrocytoma and glioblastoma (GBM) (Mann-Whitney test, p > 0.05). (**d**) Kaplan-Meier overall survival curves according to low and high *MT3* gene expression groups (log-rank test: χ^2^ = 5.694, df = 1, p = 0.017). The middle line on the scatter plot graph represents the median value, whiskers – the lowest and the highest values. Graphs symbols represent different astrocytoma grade: grade I astrocytoma – grey rhombus, grade II astrocytoma – black round, gemistocytic astrocytoma - red dot, grade III astrocytoma – grey triangle, GBM - black rectangular. Gene expression level presented as log_2_(2^−ddCt^).

The analysis of *MT1A, MT1E, MT1X, MT2, MT3* gene mRNA expression was determined in n = 51, n = 53, n = 49, n = 55, n = 51 glioma patients respectively. First, we analysed *MT* genes mRNA expression between astrocytic gliomas – grade I-III astrocytoma and grade IV astrocytoma, or glioblastoma. Analysis showed the trend for increasing *MT1A, MT1E, MT1X, MT2* and *MT3* gene expression in higher malignancy tumours, e.g. glioblastomas as compared to I-III grade astrocytomas (Figs 1a,c,e, 2a,c). However, only *MT1A* and *MT2* expression reached a statistically significant level (Mann-Whitney test, p < 0.05), while *MT1E* and *MT3* genes showed the tendency of higher expression in glioblastomas (Mann-Whitney test, p = 0.120, p = 0.058 respectively). It is interesting to notice, that grade I-III astrocytoma group consisted of one gemistocytic astrocytoma sample for which individual data dots plotted for *MT1A, MT1E, MT2*, and *MT3* genes in mRNA expression plots were the largest observations reaching the upper whiskers value which corresponded to the 3-rd quartile of gene expression value in glioblastoma, respectively (Figs 1a,c, 2a,c, sample log_2_ (2^−ddCt^) value marked as a red dot). Gemistocytic tumour is a rare variant of astrocytoma grade II with the tendency quickly develop to anaplastic astrocytoma or glioblastoma with poor prognosis^17,18^.

Next, gene expression data were grouped into “low” and “high” gene expression groups according to median values, and further *MT* gene mRNA expression associated with tumour malignancy grade was analysed. It was shown that higher percentage of glioblastomas fall into *MT* high expression group (from 37.7% for *MT1E* to 43.6% for *MT2*) as compared to grade I-III astrocytomas (from 5.5% for *MT2* to 13.2% for *MT1E*). However, only the *MT2* gene reached statistically significant difference (Chi-square test, p < 0.05).

### *MT1A* gene methylation decreases in glioblastoma

**Figure 3:**
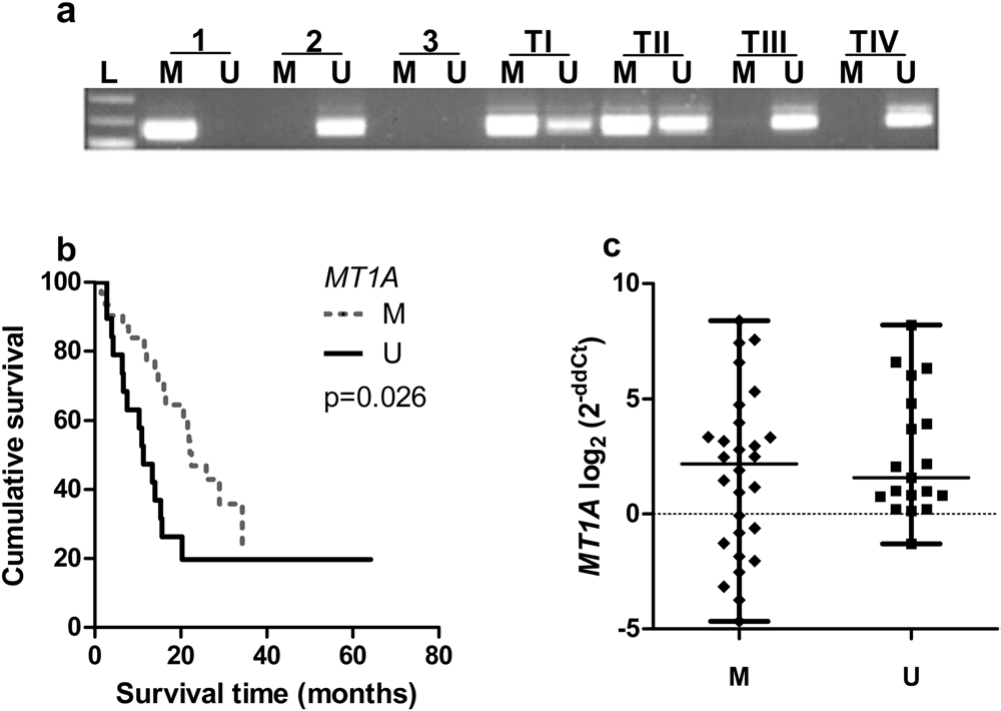
Methylation analysis of the *MT1A* gene. (**a**) Representative electrophoregram of *MT1A* gene MS-PCR. L-molecular weight marker, M- primer for methylated DNA, U-primer for unmethylated DNA, 1- methylated DNA control, 2- unmethylated DNA control, 3 - water control, TI-TIV- tumour samples. TI-TII samples estimated as methylated gene promoter while TIII, TIV as unmethylated gene promoter. (**b**) Kaplan-Meier overall survival curves according to the methylation status of *MT1A* gene. M – methylated, U – unmethylated. Unmethylated gene promoter is associated with shorter patients survival (log-rank test: χ^2^ = 4.948, df = 1, p = 0.026). (**c**) mRNA expression of *MT1A* gene in methylated (M) and unmethylated (U) promoter status groups (Mann-Whitney test, p > 0.05). The middle line on the graph represents a median value, whiskers – the lowest and the highest values.

The analysis of *MT1A* gene promoter methylation using MS-PCR was determined in 50 out of 55 of the glioma patient samples used for gene expression analysis. The methylation status was estimated according to the signal appearing on a gel. In the case of methylated or both signals, the gene was designated as methylated (Fig. 3a). Our analysis showed that the *MT1A* gene was methylated in 62% (31/50) of gliomas. The highest methylation frequency was determined in grade II-III astrocytoma (from 66.7% to 83.3%) and the lowest – in glioblastoma (57.6%). The association analysis between *MT1A* gene methylation status and patients gender showed no statistically significant differences (Chi-square test, p > 0.05) (data not shown). We looked for *MT1A* gene promoter methylation associated with gene expression. The association analysis between *MT1A* gene mRNA expression and gene promoter methylation status was not statistically significant (Chi-square test, p > 0.05) (Fig. 3c).

### *MTs* affects on patient survival

For Kaplan-Meier analysis, *MT* gene’s “low” and “high” expression groups were utilised to compare survival curves. Significant relation was shown between *MT1A, MT1X, MT2* and *MT3* genes and survival time when high gene expression was associated with poor patients survival (log-rank test for *MT1A*: χ^2^ = 6.400, df = 1, p = 0.011; *MT1X*: χ^2^ = 4.672, df = 1, p = 0.031; *MT2*: χ^2^ = 8.542, df = 1, p = 0.003; *MT3*: χ^2^ = 5.694, df = 1, p = 0.017). *MT1E* higher gene expression showed the tendency to be associated with worse patient outcome (log-rank test: χ^2^ = 3.410, df = 1, p = 0.065) (Figs 1b,d,f, 2b,d). Median survival time within high gene expression group was 11.3 months for *MT1A, MT1X* genes, 11.5 months for *MT1E, MT2* and *MT3* genes, while in low expression group 21.6 months for *MT1A, MT1E* genes, 25.9 months for *MT1X*, 28.98 months for *MT2* and *MT3* genes. Although, Kaplan-Meier analysis for survival curves in patient groups having tumours with methylated and unmethylated *MT1A* gene was performed. The statistical analysis showed significant survival difference when unmethylated gene promoter was associated with poor patients survival (log-rank test: χ^2^ = 4.948, df = 1, p = 0.026) (Fig. 3b). Median survival time within methylated gene promoter patient group was 22.47 months while within unmethylated 11.27 months.

## Discussion

In this study, we performed a thorough analysis of metallothionein gene activity in I to IV grade astrocytomas. Here we checked *MT1A, MT1E, MT1X, MT2, MT3* gene expression level and epigenetic regulation for one of the highly expressed metallothioneins, *MT1A*, in different grade gliomas and looked for clinical significance of *MT* gene activity in glioma tissue in terms of patient survival. We distributed our study into three steps – determination of *MT* genes expression in I-IV grade glioma, thorough analysis of *MT1A* gene determining expression regulation through promoter methylation and clinical importance of *MT* gene activity.

We analysed mRNA expression level and methylation status association with patient gender and age. Analysis for *MT1A, MT2, MT3* showed associations with female gender and patient age older than 50 years. We assume, that those gender associations could be due to unequal gender distribution between different malignancy groups while patients within glioblastoma group gathered in a study period comprised mainly women. Likewise, we determined that the methylated *MT1A* gene promoter was associated with younger patient age (37.1% vs. 27.4%, respectively). To our knowledge, there are no reports about *MT* promoter methylation associations with age. As glioblastoma develop more frequently in older patients, we simulate that unmethylated promoter was more frequently observed in glioblastomas as compared to I-III astrocytomas due to the patient age distribution differences.

Next, *MT* gene mRNA analysis showed an association between higher gene expression value and more aggressive tumour type, in particular, GBM. Furthermore, survival analysis revealed poor prognosis for patients having high gene expression. Our results are in accordance with Shai-Mehrian *et al*. which analysed 67 glioblastoma samples and determined *MT* gene expression, including *MT1A, MT1E, MT3* increase in glioblastoma and association with poor patients survival^16^. Up-regulation of *MT* genes are also reported for other types of cancer, for example, breast, renal or bladder^15,19,20^. According to obtained results, increased *MT* expression is closely associated with higher malignancy grade of astrocytic gliomas. Expression of metallothionein genes is inducible by a number of cell harmful agents such as heavy metals, as well as oxidative stress^8,21^. Our results suggest that *MT1A* and *MT2* genes could be used as markers for malignancy of astrocytic gliomas. While, *MT1A, MT1X, MT2, MT3* genes could be used to prognosticate a patient’s survival.

Epigenetic dysregulation is associated with various tumours, including gliomas^22^. One of the proposed *MT* genes expression regulation mechanisms is epigenetic^9^. *MT* genes play a crucial role in many solid tumours and the importance of epigenetic modifications of *MT* genes should be more analysed. Moreover, it is known that hypermethylation of specific CpG regions in *MT* genes could be used as a prognostic and diagnostic marker for some tumours, for example, *MT1M* and *MT1G* in hepatocellular carcinoma (HCC)^23^. Also, it is suggested that methylation of *MT1* gene cluster (*MT1F, MT1M*) is associated with the gene silencing and oncogenic process in breast cancer^24^. In our study, methylation analysis of *MT1A* gene revealed gene promoter methylation in 57.6% of glioblastoma while methylation in grade III astrocytoma reached 83.3%. These findings suggest that *MT1A* gene could act as an oncogene because of *MT1A* gene up-regulation and progressively apparent unmethylation in glioblastoma. This is the opposite to Yu *et al*. who performed CpG island methylation analysis for *MT1A* gene using conventional PCR in astrocytic gliomas. The Yu group determined no statistically significant *MT1A* gene methylation differences between I-IV grade astrocytoma, while a slight increase in methylation abundancy was detected in glioblastoma (21.4%, 33%, 25.1% and 41.6% for I-IV grades respectively)^21^. These discrepancies suggest that the implication of *MT* gene hypermethylation in astrocytic glioma pathology deserves further analysis.

In conclusion, this study is one of the most detailed analyses of *MT* genes expression in different grade glioma. Our study suggests that *MT* genes expression is associated with tumour malignancy grade and higher gene expression level is more expected in grade IV astrocytoma (glioblastoma). Furthermore, *MT1A* gene expression and methylation analysis showed that shorter patient survival is strongly associated with unmethylated and highly expressed gene.

## Methods

### The subject

There were investigated 55 post-operative tissues of patients with different grade gliomas: pilocytic astrocytoma, grade I (n = 2), diffuse astrocytoma, grade II (n = 8), gemistocytic astrocytoma, grade II (n = 1), anaplastic astrocytoma, grade III (n = 6), glioblastoma (GBM), grade IV (n = 38). All the tissues were surgically resected and gathered at the Department of Neurosurgery of the Lithuanian University of Health Sciences, Kaunas Clinics, Lithuania, from 2014 to 2016. The patient samples were diagnosed according to the histopathology test results and WHO classification at the Department of the Pathological Anatomy of the Lithuanian University of Health Sciences. All patients underwent standard treatment, comprised of resection followed by radiotherapy plus concurrent temozolomide (TMZ) chemotherapy. All study methodologies were performed in accordance with the regulations and guidelines and were approved by the Regional Bioethics Committee of the Lithuanian University of Health Sciences, Kaunas, Lithuania (No. P2-9/2003). All patients gave their written informed consent before surgery.

For the *MT* gene expression and methylation analysis in total 55 samples were analysed as follows: I-III malignancy grade astrocytic gliomas (n = 17) and GBM (n = 38). The grade I-III astrocytic gliomas group comprised of 88.2% (n = 15) men and 11.8% (n = 2) women. The patient age ranged from 21 to 63 yr and the median age was 32 yr. The median survival time was 27 months and ranged from 10.3 to 64.2 months. The GBM group comprised of 47.4% (n = 18) men and 52.6% (n = 20) women. The age of GBM patients ranged from 36 to 82 yr and the median age was 61 yr. The median survival time was 11.8 months and ranged from 1.2 to 34.6 months.

### Quantitative real-time polymerase chain reaction (qRT-PCR) with SYBR Green Dye

Total RNA was extracted from 50–100 mg of tumourous tissue with “*mir*Vana miRNA Isolation Kit” (ThermoFisher Scientific, USA). cDNA synthesis was made of 2 μg of total RNA with “High-Capacity cDNA Reverse Transcription Kit“ (Applied Biosystems, USA). The mRNA expression level of *MT* genes (*MT1A, MT1E, MT1X, MT2, MT3*) and *β-actin* as reference gene were detected with qRT-PCR with SYBR Green dye using real-time PCR system “AB 7500 Fast” (Applied Biosystems, USA). The reaction was performed in 12 µl of the total volume of mixture which included 6 µl Maxima SYBR Green/ROX qPCR Master Mix (2x) (ThermoFisher Scientific, USA), 15 ng cDNA, water, nuclease-free and gene primers, with the concentration of 0.2 µM for *MT1A, MT1E, MT1X* and 0.1 µM for *MT2, MT3*, and *β-actin. MT* gene-specific primers were used as follows: 5′-CTTGGGATCTCCAACCTCAC-3′ (forward), 5′-AGGAGCAGCAGCTCTTCTTG-3′ (reverse) for *MT1A* gene; 5′-GGGCTCCATTCTGCTTTCCA-3′ (forward), 5′-TTGGGGTCCATTTCGAGCAA-3′ (reverse) for *MT1E* gene; 5′-CTGCTTCTCCTTGCCTCGAA-3′ (forward), 5′-TGTCTGACGTCCCTTTGCAG-3′ (reverse) for *MT1X* gene; 5′-ATCCCAACTGCTCCTGCGCCG-3′ (forward), 5′-CAGCAGCTGCACTTGTCCGACG-3′ (reverse) for *MT2* gene; 5′-CTGAGACCTGCCCCTGCCCTT-3′ (forward), 5′-TGCTTCTGCCTCAGCTGCCTCT-3′ (reverse) for *MT3*^25^. *MT1A* gene expression primers were designed using Primer 3^26^. As an endogenous control *β-actin* gene was used with the primer sequences published^27^: 5′-AGAGCTACGAGCTGCCTGAC-3′ (forward), 5′-AGCACTGTGTTGGCGTACAG-3′ (reverse). The amplification cycling conditions included: the initial denaturating at 95 °C for 10 min, 40 cycles at 95 °C for 15 s, annealing at 60–68 °C (for *MT1A, MT1E, β-actin* 60 °C, for *MT1X* 66 °C, for *MT2, MT3* 68 °C) for 30 s, 72 °C for 30 s and the final stage for melting curve at 95 °C for 15 s, 60 °C for 1 min, 95 °C for 15 s, 60 °C for 15 s. Each tumour sample was analysed in triplicates. *MT* gene expression was normalized to healthy human brain RNA samples (RHB) “Total RNA-Human Adult Normal Tissue 5 donor Pool: Brain“ (BioChain) using 2^−ΔΔCt^ method where ΔΔCt = ΔCt_tumour tissue_ (Ct_MT_ − Ct_*β-actin*_)− ΔCt_RHB_ (Ct_MT_ − Ct_*β-actin*_). For the graphical representation, the log_2_ scale was used.

### Methylation-specific polymerase chain reaction (MS-PCR)

For the *MT1A* gene methylation analysis DNA was extracted from 50–100 mg of tumourous tissue samples by salting-out method using 10% SDS (Invitrogen, USA), proteinase K (ThermoFisher Scientific, Germany), followed by Trichlormetan/Chloroform (Carl Roth^®^ GmbH, Germany) and 6 M sodium chloride (Carl Roth^®^ GmbH, Germany) extraction and DNA precipitation with 96% ethanol. A total of 400 ng of extracted DNA was treated with sodium bisulfite using “EpiJET Bisulfite Conversion Kit“ (ThermoFisher Scientific, USA) according to the manufacturer’s protocol. The methylation status of the *MT1A* gene was determined by MS-PCR. The pair of primers were designed using “MethPrimer“ tool^28^. The primers for methylated *MT1A* allele 5′-TTTACGTTTTGTATTACGTCGATTC-3′ (forward) and 5′-CAACCTTAACGTTTCGATCG-3′ (reverse), PCR amplicon size 127 bp and for unmethylated allele 5′-TATGTTTTGTATTATGTTGATTTGG-3′ (forward) and 5′-CCAACCTTAACATTTCAATCACC-3′ (reverse), PCR amplicon size 126 bp (Metabion International AG, Germany). The reaction was performed in 15 µl of the total volume which comprised of 7.5 µl Maxima Hot Start Green PCR Master Mix (2x) (ThermoFisher Scientific, USA), 1 µM of each primer, 1 µl bisulfite treated DNA and nuclease-free water. The cycling conditions included: the initial denaturation at 95 °C for 5 min, 38 cycles at 95 °C for 15 s, 60 °C for 30 s, 72 °C for 30 s and 72 °C for 5 min. The reaction was performed with “Veriti 96 Well Thermal Cycler” (Applied Biosystems, Singapore). After PCR, the amplicons were separated on 2% agarose gel and visualized under UV gel imaging system (GelDoc XR System, BioRad, USA).

### Statistical analysis

The statistical analyses were performed by SPSS 22 and GraphPad Software “Prism 5” programmes. Nonparametric Mann-Witney test was used to compare continuous variables in two different variable groups (tumour grade groups, promoter methylation groups). The Chi-square test was used to analyse the association between mRNA expression, methylation status, and patients clinical characteristics. Kaplan-Meier analysis (Log-rank test) was used to determine the differences between patient survival curves in different gene expression or gene methylation groups. The p < 0.05 value was considered statistically significant.

## Data Availability

The datasets generated and analysed in the current study are available under request from the corresponding author.
